# Nutrition, health benefits, and processing of sand rice (*Agriophyllum squarrosum*): Comparisons with quinoa and buckwheat

**DOI:** 10.1002/fsn3.4429

**Published:** 2024-08-27

**Authors:** Xiaofan Yang, Wenting Fu, Liuyang Xiao, Zhaojun Wei, Lihong Han

**Affiliations:** ^1^ The Collaborative Innovation Center for Food Production and Safety, College of Biological Science and Engineering North Minzu University Yinchuan Ningxia China

**Keywords:** buckwheat, health benefit, processing, quinoa, sand rice

## Abstract

The dual pressures of climate change and population growth have made the development of new grains a necessity. *Agriophyllum squarrosum* (sand rice) has high adaptability to harsh environments and does not occupy agricultural land. It is widely cultivated and consumed in Central Asia. Sand rice, together with quinoa and buckwheat, belongs to the same pseudocereals group with rich nutritional value and gluten‐free properties; however, its nutritional composition and health benefits differ from those of quinoa and buckwheat. Sand rice seeds are a rich source of nutrients and bioactive compounds, including proteins, amino acids, unsaturated fatty acids, and crude fiber, which are similar to those in buckwheat and quinoa; however, their starch content is relatively low. Sand rice seeds also possess phenolic acids and flavonoids, which exhibit antioxidant, anticancer, anti‐diabetes, and anti‐inflammatory properties. Furthermore, sand rice extracts are considered suitable for treating some chronic diseases. Overall, sand rice is considered a good plant‐based food that can be used to develop various functional foods and beverages or mixed with other grains in different recipes. However, advancements in the processing technology of sand rice‐based foods are required to fully exploit the potential of sand rice in the food industry to improve human health. This review analyzes the current understanding of the nutritional content of sand rice by comparing it with that of quinoa and buckwheat. Furthermore, its potential medicinal activity and feasibility as a functional ingredient to improve food quality is discussed.

## Graphical abstract

In this review, we compare the properties of Agriophyllum squarrosum (sand rice), which is known for its rich nutritional content, strong drought adaptability, and therapeutic potential, with quinoa and buckwheat to provide a comprehensive understanding of its feasibility as a functional ingredient to improve food quality.

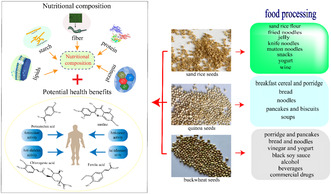

## 1 INTRODUCTION

Climate change has affected the production of multiple crops worldwide. Concurrently, sustained population growth has increased the demand for food production (Lyzenga et al., 2021). Consequently, there is an urgent necessity to cultivate crops that can withstand harsh conditions to meet the growing food requirements. Sand rice (*Agriophyllum squarrosum*) is an annual psammophyte commonly grown in arid and semi‐arid regions; it is mainly distributed in desert regions or barren lands across countries such as China, Mongolia, Russia, and Iran (Liu, Li, et al., 2023; Liu, Shen, et al., 2023). The morphological and physiological characteristics of sand rice, including well‐developed roots and thick stems, confer excellent resistance to drought, cold, and heat (Chen et al., 2014). Furthermore, it has strong vitality and adaptability to climate change and extreme weather (Xu et al., 2020).

As early as the Tang Dynasty, sand rice seeds were used as food rations for soldiers; the local people in the Dunhuang area still consume sand rice seeds (Zhao et al., 2016). Under natural conditions, the seed yield of sand rice ranges from 21 to 66 kg/ha; the yield of wild sand rice millet in the Tengger Desert can reach 1281 kg/ha (Chen et al., 2014; Zhao et al., 2016). In recent years, the large‐scale cultivation of sand rice has been carried out in the Ulan Buh desert, with an average yield of 225–375 kg/ha (Zhao et al., 2021). Given its unique characteristics, the availability of farmlands in the desert area, and the lack of interference with the cultivation of other common crops, sand rice has emerged as an important food crop. It is a potential novel future food that can alleviate the impending food production crisis and facilitate developing functional foods (Chen et al., 2014).

Sand rice has a balanced and comprehensive nutritional value and is rich in polyunsaturated fatty acids, which have significant potential in treating cardiovascular diseases, insulin sensitivity, and immunity (Xu et al., 2020). Sand rice has a broad amino acid profile, including all essential amino acids that promote bone growth and enhance specific human immunity, making it an excellent health food (Zhao et al., 2021). Moreover, sand rice seeds contain selenium, iron, zinc, and other trace elements and bioactive compounds that possess health‐benefitting properties. These bioactive compounds, including flavonoids, chlorogenic acid, and phenolics, are potential therapeutic agents for diabetes, cancer, inflammation, hyperlipidemia, and other diseases (Wang, Gong, et al., 2020; Wang, Wang, et al., 2020). Sand rice and quinoa belong to the subfamily Chenopodioideae, and they lack the botanical characteristics of ordinary grains such as wheat and corn. Therefore, they are classified as pseudocereals (Chen et al., 2014; Hussain et al., 2021). In recent years, pseudocereal grains, specifically quinoa (*Chenopodium quinoa* Willd) and buckwheat (*Fagopyrum esculentum*), have garnered significant attention owing to their rich nutritional value and gluten‐free properties (Martínez‐Villaluenga et al., 2020).

However, owing to an insufficient understanding of the health benefits of sand rice, the current consumption of sand rice is rather limited; furthermore, compared to quinoa and buckwheat, sand rice is not well researched. Therefore, the aim of this study was to comprehensively review the nutritional composition and value of sand rice, compare it with those of quinoa and buckwheat, and summarize the latest advances in sand rice processing technologies and its health benefits.

### 1.1 Nutritional composition of sand rice, quinoa, and buckwheat

The nutritional value of sand rice is generally higher than that of other grains such as corn, wheat, and rice. It has a high‐protein, crude fiber, and fat content, along with a wide amino acid spectrum. The amino acid profile of sand rice presents a new resource for safe and pollution‐free high‐protein food for humans (Wang et al., 2019). Quinoa and buckwheat are healthy gluten‐free foods. Compared to other ordinary grains, quinoa, and buckwheat have higher nutritional value and are rich in protein, starch, fiber, and various bioactive substances (Christa & Soral‐Śmietana, 2008; Nowak et al., 2016). A comprehensive evaluation of the nutritional value of sand rice, quinoa, and buckwheat is given in Table 1 to enable a comprehensive understanding of sand rice.

**TABLE 1:** Nutritional composition of sand rice, quinoa, and buckwheat grains.

Grain	Components	Value	References
Sand rice	Carbohydrates (% dry basis)	45	Chen et al. (2014)
		32.5–51.2	Xu et al. (2020)
	Starch (% dry basis)	32.5–51.2	Cao et al. (2022)
		48.8	Peng et al. (2018)
	Crude fiber (% dry basis)	8.6	Zhao et al. (2016)
		4.9–14.9	Xu et al. (2020)
	Protein (g/100 g protein)	23.3	Zhao et al. (2016)
		21.6–25.5	Xu et al. (2020)
		24.16	Wang et al. (2019)
	Lipids (% dry basis)	7.7–11.8	Xu et al. (2020)
		10.61	Wang et al. (2019)
		6.0–74.7	Mohamed Ahmed et al. (2021)
Quinoa	Carbohydrates (% dry basis)	48.5–69.8	Pathan and Siddiqui (2022)
		48.5–69.8	Nowak et al. (2016)
		59.9–74.7	Vilcacundo and Hernández‐Ledesma (2017)
		67–74	Hussain et al. (2021)
		57.19	Li, Lietz, and Seal (2021); Li, Ye, et al. (2021)
		32–69	Vilcacundo and Hernández‐Ledesma (2017)
	Starch (% dry basis)	64.2	Mohamed Ahmed et al. (2021)
		10	Nowak et al. (2016)
	Total dietary fiber (% dry basis)	7.0–11.7	Vilcacundo and Hernández‐Ledesma (2017)
		11.10	Ren et al. (2022)
		9.53	Li, Lietz, and Seal (2021); Li, Ye, et al. (2021)
		8.61	Ren et al. (2022)
	Crude fiber (% dry basis)	1.81–6.51	Hussain et al. (2021)
		7.0–11.4	Pathan and Siddiqui (2022)
		11.8–14.66	Li, Lietz, and Seal (2021); Li, Ye, et al. (2021)
	Protein (g/100 g protein)	8–22	Mohamed Ahmed et al. (2021)

		7	Nowak et al. (2016)
		16.70	Ren et al. (2022)
		13.1–16.7	Vilcacundo and Hernández‐Ledesma (2017)
		10–18	Hussain et al. (2021)
		9.1–15.7	Pathan and Siddiqui (2022)
		4.8–7.5	Li, Lietz, and Seal (2021); Li, Ye, et al. (2021)
	Lipids (% dry basis)	1.8–10.0	Mohamed Ahmed et al. (2021)
		7	Nowak et al. (2016)
		5.5–7.4	Vilcacundo and Hernández‐Ledesma (2017)
		4.4–8.8	Hussain et al. (2021)
		4.0–7.6	Pathan and Siddiqui (2022)
	59–70	Bertazzo et al. (2011)
Buckwheat	Carbohydrates (% dry basis)	46.70–56.62	Neacsu et al. (2022)
		70	Gao et al. (2020)
		3.6–10.6	Lu et al. (2013)
	Starch (% dry basis)	7	Ahmed et al. (2014)
	Total dietary fiber (% dry basis)	2.9–34.4	Islam et al. (2023)
		11.71–12.24	Neacsu et al. (2022)
	Protein (g/100 g protein)	10.2–17.9	Lu et al. (2013)
		12	Ahmed et al. (2014)
		0.2–10.1	Islam et al. (2023)
		4	Ahmed et al. (2014)
	Lipids (% dry basis)	1.44–1.71	Neacsu et al. (2022)
		1.5–4	Christa and Soral‐Śmietana (2008)

#### 1.1.1 Starch

Starch is the main carbohydrate component in the endosperms of sand rice seeds, quinoa, and buckwheat grains (Peng et al., 2018; Thakur et al., 2021). The total carbohydrate content of sand rice is approximately 45% (Xu et al., 2020), of which starch accounts for 51.2%; however, the starch content of shelled sand rice seeds can exceed 70% (Cao et al., 2022; Peng et al., 2018). The starch contents of quinoa and buckwheat are relatively higher than those of sand rice, at 64.2% and 70%, respectively (Thakur et al., 2021). The total starch content in quinoa seeds is 32%–69%, whereas that in common buckwheat seeds is 22.48%–31.58% (Gao et al., 2023; Wu et al., 2017). Owing to the high content of resistant and slow digestible starches, the digestibility of sand rice is lower than that of quinoa (Han et al., 2021).

**FIGURE 1:**
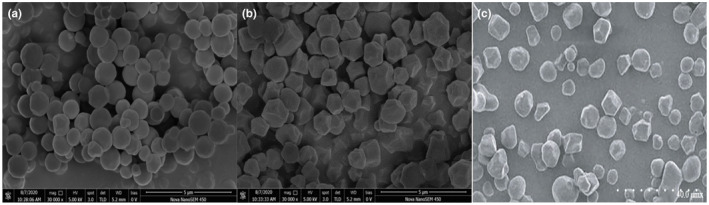
Scanning electron microscopy images of (a) sand rice starch (×30,000), (b) quinoa starch (×30,000), and (c) buckwheat starch (×10,000) (Han et al., 2021; Huang et al., 2021).

The surface of sand rice starch particles is smooth and spherical (Figure 1a), with an average particle size of 1.12–1.15 μm, categorizing them as very small among starches (Han et al., 2021). The starch particles of quinoa seeds are polygonal, with a particle size of 0.4–2 μm (Figure 1b) (Zhao et al., 2021), and are also categorized as very small‐granule starches. Buckwheat starch particles are spherical, oval, and polygonal (Figure 1c), with diameters mostly between 5.6 μm and 7.3 μm, significantly larger than those of sand rice and quinoa (Sindhu et al., 2019). The characteristics of small‐granule starches in sand rice and quinoa are advantageous in food processing and non‐food fields (Zhao et al., 2021).

The amylose content of sand rice starch is 26.31%, lower than that of cornstarch (27.71%) (Peng et al., 2018). However, one study has reported a slightly higher content of amylose (31.2%) in the endosperm starch of sand rice seeds, which may be related to the differences in sand rice varieties and measurement methods (Ren et al., 2020). The amylose content of quinoa starch is between 4.7% and 17.3%, slightly lower than that of sand rice starch (Wu et al., 2017). There are significant differences in the amylose content of buckwheat starch, varying from 15% to 52% (Gao et al., 2023; Thakur et al., 2021). According to Han et al. (2021), the X‐ray diffraction pattern of sand rice starch shows a typical A‐type crystalline structure consistent with that of quinoa and buckwheat starches. Furthermore, sand rice crystallinity is 22.00%–37.95%, which is similar to that of quinoa (21.00%–29.67%) and buckwheat (24.74%–27.19%) but higher than that of wheat and corn (Gao et al., 2020, 2023; Jiang et al., 2020; Peng et al., 2018; Ren et al., 2020).

The degree of polymerization (DP) is typically used to determine the average number of glucose units in starch molecules. The branched starch unit chains of sand rice starch mainly comprise the A‐chain (without carrying any other chains, DP 6–8), followed by the short B‐chain (DP 8–25) and fingerprint B‐chain with DP 3–7, similar to those of quinoa starch (Han et al., 2021). Furthermore, comparing the apparent average molar masses (Mw), apparent number‐average molecular weight (Mn), and polydispersity values (Mw/Mn) of starch, sand rice starch has a higher Mw (5.21 × 10^6^ g/mol–6.13 × 10^6^ g/mol) and lower Mw/Mn (1.34–1.57) than quinoa starch (Mw = 3.27 × 10^6^ g/mol, Mw/Mn = 18.2). Therefore, sand rice starch has more substitution structures than quinoa regarding the main chain or residues of the glucose group (Han et al., 2021). However, compared to buckwheat starch (Mw = 3.86 × 10^7^ g/mol–4.68 × 10^7^ g/mol, Mw/Mn = 2.67–2.72), the Mw and Mw/Mn of sand rice starch are lower, indicating that sand rice starch is composed of a branched starch with lower polymerization and possesses a more uniform molecular weight distribution (Gao et al., 2020).

The swelling power and solubility of sand rice, quinoa, and buckwheat starch show a similar trend within the same temperature range (between 55°C and 95°C). The reported gelatinization onset temperature (*T*_0_) of sand rice starch is 64–68.1°C, the peak temperature (*T*_*p*_) is 70–74.2°C, and the conclusion temperature (*T*_c_) is 78–84.1°C (Han et al., 2021; Ren et al., 2020). Upon comparing the thermal properties of the three starches, the onset, peak, and conclusion temperatures of sand rice starch were found to be higher than those of quinoa starch (*T*_0_ = 57.89–61.76°C, *T*_p_ = 63.77–67.44°C, *T*_c_ = 71.86–77.24°C, respectively) and buckwheat starch (*T*_0_ = 55.70–62.53°C, *T*_p_ = 66.85–70.45°C, *T*_c_ = 73.60–80.32°C, respectively); sand rice starch has a higher enthalpy (Δ*H* = 19.8–24.7 J/g) than quinoa starch (7.79–11.76 J/g) and buckwheat starch (6.44–8.92 J/g) (Gao et al., 2020, 2023; Han et al., 2021). Moreover, the pasting temperatures of sand rice, quinoa, and buckwheat starches are 78.32–79.17°C, 68.65°C, and 66.42–71.13°C, respectively (Gao et al., 2023; Han et al., 2021). The gelatinization temperature is positively related to the integrity of the starch crystal structure, indicating that the crystal structure of sand rice starch is more stable than that of quinoa and buckwheat starches (Gao et al., 2020). However, the peak and final viscosities of glutinous sand rice starch are lower than that of quinoa starch but higher than that of buckwheat starch, making it easier to form a gel; this property can be used to make gluten‐free bread or as a thickener for emulsified foods (salad), soups, congee, beverages, and pies. Furthermore, the breakdown value of sand rice starch (200–522 cP) is lower than that of quinoa, indicating that it can also be utilized in frozen food fields (Gao et al., 2023; Han et al., 2021).

#### 1.1.2 Fiber

Dietary fiber is a nutrient composed of non‐starch carbohydrates and lignin, which cannot be digested in the gastrointestinal tract (Turner & Lupton, 2021). Consuming appropriate amounts of dietary fiber not only improves diet quality, gastrointestinal dynamics, and food digestion but also reduces the risk of cardiovascular diseases and cancer, insulin secretion, blood cholesterol levels, and the production of enterogenic virus toxins (Snauwaert et al., 2022). The crude fiber content of sand rice is higher than that of cereals, ranging from 4.9% to 14.9% with an average of 8.6%, which is significantly higher than that of wheat flour, rice, and corn (Wang et al., 2019; Xu et al., 2020). The total dietary fiber content of quinoa and buckwheat is between 7% and 11.7% and 3.6% and 10.6%, respectively (Lu et al., 2013; Vilcacundo & Hernández‐Ledesma, 2017).

According to the water solubility, dietary fibers are usually divided into soluble and insoluble fibers (Chen et al., 2021). The soluble fiber component of quinoa is mainly composed of arabinose and homologous galacturonic acid, accounting for approximately 22% of the total fiber in quinoa seeds (Mohamed Ahmed et al., 2021; Zhu, 2020). The soluble fiber component of buckwheat seeds is mainly composed of pectin, galactose, and glucan, accounting for 62.5% of the total dietary fiber in buckwheat (Ahmed et al., 2014; Zhu, 2020). Crude fiber intake can prevent obesity and promote colon function (Wang et al., 2019). Therefore, sand rice, quinoa, and buckwheat seeds can be utilized as functional food to improve blood sugar levels and regulate body weight in patients with diabetes.

#### 1.1.3 Proteins

Studies have demonstrated the nutritional and health benefits of proteins found in plant‐based foods, such as soy protein (Ahnen et al., 2019). Sand rice has a relatively higher protein content of 25.5% than quinoa (16.70%) and buckwheat (11.71%–12.24%) (Xu et al., 2020). Moreover, sand rice, quinoa, and buckwheat are rich in various amino acids. Sand rice contains many types of amino acids, with a high content of essential amino acids. Lysine is the main amino acid in sand rice, surpassing the levels found in quinoa and buckwheat (Pathan & Siddiqui, 2022; Zhao et al., 2016). Lysine, arginine, and leucine contents in sand rice also exceed those in common grains, such as wheat, rice, and barley (Wang et al., 2019; Xu et al., 2020).

Quinoa has high contents of amino acids that are limited to wheat and corn; these include lysine, methionine, and threonine (Nowak et al., 2016). Buckwheat grains are relatively rich in glutamic acid, aspartic acid, arginine, and lysine. Furthermore, a common characteristic of sand rice, quinoa, and buckwheat is their low levels of prolamins. Prolamins (gliadin in wheat and related proteins) in grains are associated with intestinal damage reactions observed in individuals with celiac disease (Ferretti et al., 2012). The consumption of gluten‐free cereals benefits patients with celiac disease, making pseudocereals a safe alternative food for them. Compared to quinoa and buckwheat, sand rice offers amino acid types and contents that can fully meet the dietary amino acid requirements of adult humans without the need for additional food supplementation (Xu et al., 2020; Zhao et al., 2021). Therefore, sand rice has a huge potential in complementing the nutritional deficiencies of typical gluten‐free foods along with quinoa and buckwheat (Graziano et al., 2022).

#### 1.1.4 Lipids

Unsaturated fatty acids have several health benefits for the human body, and consuming them can help prevent oxidative stress, cancer, and cardiovascular and cerebrovascular diseases (Liu, Li, et al., 2023; Liu, Shen, et al., 2023). Common examples of unsaturated fatty acids include oleic, linoleic, and linolenic acids. The lipid content of sand rice ranges between 9.7% and 15.14%, significantly higher than that of quinoa and buckwheat; sand rice is also rich in linoleic (67.42%), oleic (16.69%), and linolenic acids (4.21%) (Xu et al., 2020). In quinoa, unsaturated fatty acids account for 88% of the total fatty acids and consist primarily of oleic (19.97%–22.2%), linoleic (59.35%–71.87%), and linolenic acids (3.54%–6.21%), similar to those in soybean oil (Shen et al., 2022). Quinoa seeds are a major source of essential fatty acids and can be viable alternatives to oilseeds (Mohamed Ahmed et al., 2021).

The total fat content of buckwheat grains is between 2.8% and 3.4%, with oleic, linoleic, and palmitic acids being the main fatty acids (Golijan et al., 2019). Among these, oleic acid constitutes the highest proportion (53.42%), followed by palmitic acid (18.6%) and linoleic acid (12.04%) (Golijan et al., 2019). Unsaturated fatty acids account for most of the oil content of sand rice seeds, emphasizing their broad application prospects. Its higher linoleic acid content, compared to that of quinoa and buckwheat, makes sand rice particularly effective in preventing and treating hyperlipidemia, hypertension, arteriosclerosis, and coronary heart disease (Zhao et al., 2016).

#### 1.1.5 Minerals

Trace elements are present in very low concentrations in the body and are closely related to the metabolism and physiological processes (Islam et al., 2023). The ash content of sand rice is approximately 5%, higher than that of quinoa and buckwheat (Zhao et al., 2016). Sand rice is rich in calcium, iron, and zinc, which are necessary for the human body. The regular consumption of sand rice is beneficial to bone health and alleviation of iron‐deficiency anemia. Phosphorus is an essential element involved in various physiological processes, and together with calcium, it is a key component of bones. However, the phosphorus content of sand rice is significantly lower than that of quinoa and buckwheat (Mohamed Ahmed et al., 2021; Sinkovič et al., 2022; Wang et al., 2019). Therefore, when phosphorus supplement is required, quinoa and buckwheat can be selected for consumption.

Quinoa has a higher mineral content than common grains, including wheat, corn, and rice (Nowak et al., 2016). Its total mineral content is 3.4%, and its seeds are rich in calcium, magnesium, iron, copper, potassium, zinc, and other nutrients (Mohamed Ahmed et al., 2021). Among these, calcium, magnesium, and potassium exist in bioavailable forms in quinoa, and their contents are suitable for achieving a balanced diet (Vilcacundo & Hernández‐Ledesma, 2017). Buckwheat grains contain 2.0%–2.5% minerals, mostly located outside the seeds and shells. Compared to other common grains, such as rice and corn, buckwheat contains more trace elements, such as zinc, copper, and manganese (Ahmed et al., 2014), making it an important trace element source (Christa & Soral‐Śmietana, 2008).

### 1.2 Phytochemicals

The aboveground part of sand rice is recorded as a Mongolian medicine, and its seeds have medicinal effects such as clearing heat, detoxification, diuresis, and expelling plague (Xu et al., 2020). Sand rice has been reported to contain triterpenoids, steroids, alkaloids, coumarins, phenols, and flavonoids, which have the effects of invigorating the spleen and stomach, lowering blood sugar, protecting the liver, and lowering blood lipids (Xu et al., 2020). Terpenoids are a large class of secondary metabolites in plants, with anticancer, anti‐inflammatory, antiviral, and other biological activities, and can be further divided into monoterpenes, sesquiterpenes, diterpenes, triterpenoids, and tetraterpenes (Darshani et al., 2022). Two previous studies (Gong et al., 2012; Liu et al., 2013) have isolated and identified six triterpenoids from sand rice plants. Kong et al. (2018) isolated four oleano‐type triterpenoid saponins from the aerial parts of sand rice. The specific expression of the genes encoding terpenoids supported the specific enrichment of terpenoids in the aboveground tissues of sand rice (Yin et al., 2021). Based on non‐targeted metabolomics analysis, Yin et al. (2021) found that there were eight terpenoids enriched in the aboveground tissues of sand rice, among which diosgenin was the most abundant; sabinene, 18‐nor‐4,15‐dihydroxyabieta‐8,11,13‐trien‐7‐one, and polypodine B were significantly enriched in the stem and spike, respectively. In quinoa, monoterpenes and triterpenoids are the main terpenoids (Yang et al., 2022). At least 15 monoterpenoids have been identified in quinoa essential oil from the Eastern Mediterranean, and most of the triterpenoid compounds in quinoa are pentacyclic triterpenoids in the form of saponins (Lin et al., 2019). To date, 87 different saponins have been identified in quinoa seeds, mainly distributed in the bran (Martínez‐Villaluenga et al., 2020). Seven triterpenoids were isolated from three different *Fagopyrum* plants, mainly from the seed oil of buckwheat and tartary buckwheat (Huda et al., 2021).

Phytosterols are natural compounds, and a moderate daily intake (1.5–2.4 g/day) of phytosterols can prevent human disease and reduce the absorption of dietary cholesterol without harmful side effects (Jayaraman et al., 2021). Phytosterols are also found in sand rice plants. Gong et al. (2012) isolated five compounds from sand rice plants, namely spinasterol, Δ^7^‐stigmasterenol, ergost‐7,24(28)‐diene‐3‐ol, stellasterol, and cholester‐7‐en‐3β‐ol. In contrast, quinoa and buckwheat mainly contain Δ^5^‐sterols, including β‐sitosterol, campesterol, and stigmasterol (Martínez‐Villaluenga et al., 2020).

Three coumarin compounds, two alkaloid compounds, and other compounds such as fatty acids have been isolated from the sand rice plant (Gong et al., 2012; Li et al., 2012; Zhou et al., 2012). The isolation and identification of these bioactive substances support the pharmacological role of sand rice as a Mongolian medicine and provide a relevant theoretical basis for the relationship between the biological activity of sand rice and its chemical components and the future development of its efficacy.

### 1.3 Biological attributes and health benefits

**FIGURE 2:**
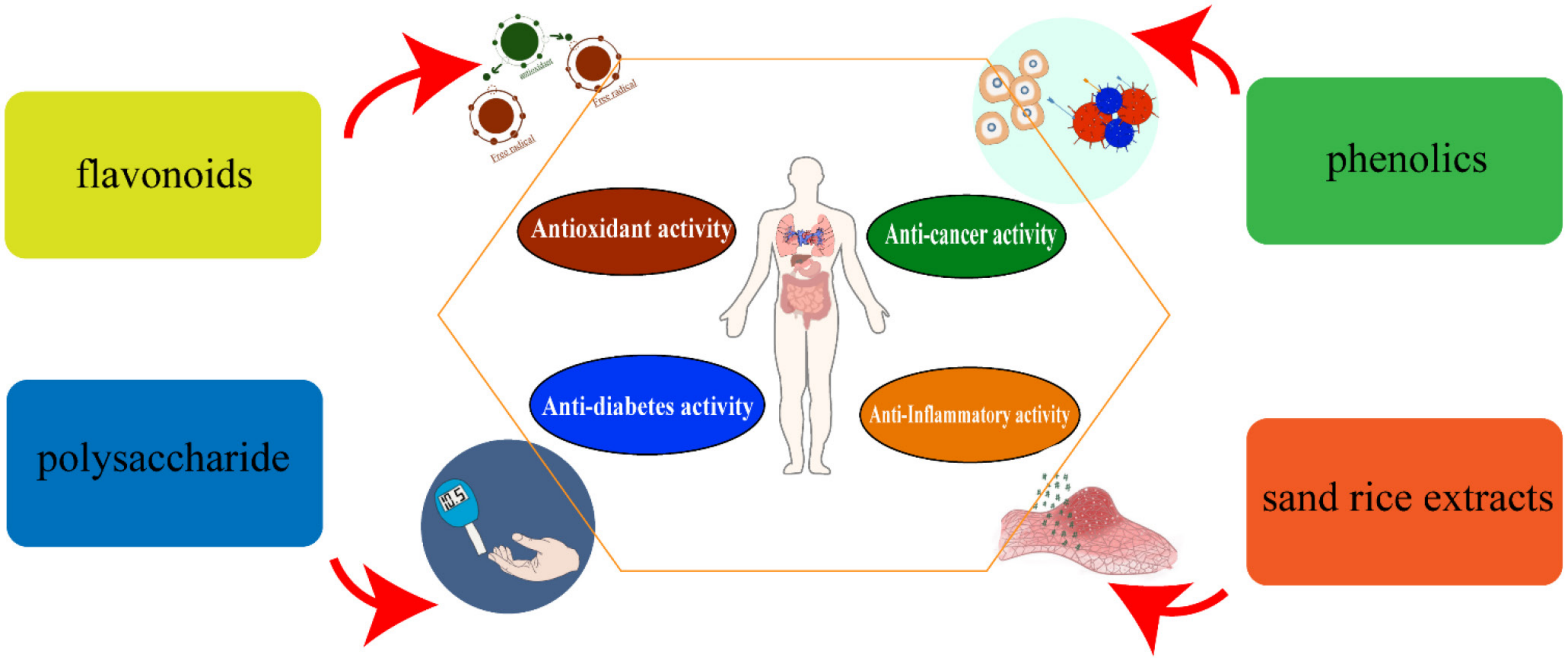
Health benefits of sand rice, quinoa, and buckwheat components.

Bioactive compounds are secondary plant metabolites with therapeutic potential. Quinoa‐ and buckwheat‐based products effectively prevent chronic diseases (Chen et al., 2018; Hussain et al., 2021). Sand rice, quinoa, and buckwheat contain many bioactive substances, such as phenols and flavonoids, which have greater health benefits than those of common grains (Figure 2). The bioactive substances and their functional activities of sand rice, quinoa, and buckwheat are listed in Table 2.

**TABLE 2:** Bioactive substances and functional activities of sand rice, quinoa, and buckwheat.

Functional activity	Grain	Compounds	Specific types
Antioxidant activity	Sand rice	Phenolic	Protocatechuic acid, Chlorogenic acid
		Flavonoids	Isoquercitrin, Luteolin‐6‐glucoside
	Quinoa		
	Buckwheat	Flavonoids	Rutin, Quercetin, Sorbetol‐3‐O‐robutin
		Proteins	
Anticancer activity	Sand rice	Phenolic	Protocatechuic acid, ferulic acid
		Flavonoids	Quercetin
	Quinoa	Amaranthin	
		Apigenin II	
		Phenolic	Erulic acid, Sorbic acid
		Polysaccharide	
	Buckwheat	Buckwheat bran extract	
		Polysaccharide	
		Micronutrients	Zinc, Selenium, Cadmium
Anti‐diabetes activity	Sand rice	RS starch, SDS starch	
		Flavonoids	Rutin
		Crude oligosaccharide	
		Sand rice ethanol extract	
Anti‐inflammatory activity	Sand rice	Sand rice extract	Water extract, AOS
	Quinoa	Lunasin	
		Protein	Glycosylation quinoa protein
		Saponin	
		Chenopodin	
	Buckwheat	Flavonoids	Rutin
		Extract of buckwheat sprouts	
		Polysaccharides	

#### 1.3.1 Antioxidant activity

Oxidative stress occurs due to an imbalanced redox system that leads to excessive free radicals and oxidants and can cause various chronic diseases (Saleem et al., 2020). The antioxidant activity of phenolic compounds in pseudocereal grains plays an important role in removing free radicals, promoting health, and preventing diseases. Assays based on 2,2′‐azino‐bis(3‐ethylbenzothiazoline‐6‐sulfonic acid) (ABTS) and 2,2‐diphenyl‐1‐picrylhydrazyl (DPPH) help to assess free radical‐scavenging abilities and iron‐reducing antioxidant activities and determine the antioxidant capacity of grains (Xiong et al., 2019). Among the compounds present in sand rice seeds, protocatechuic acid has remarkable efficacy in removing DPPH free radicals and reactive oxygen species in cells, establishing it as the most potent active antioxidant (Xu et al., 2018). The antioxidant capacity of protocatechuic acid may be related to the phenolic groups in its structure, and the main mechanism of its antioxidant activity is to trap free radicals (Reis et al., 2010). Meanwhile, protocatechuic acid shows a protective effect on t‐BOOH‐induced HepG2 damage and oxidative stress damage caused by Parkinson's disease (Xu et al., 2018). Moreover, chlorogenic acid in sand rice seeds exhibits superior potassium iron cyanide reduction abilities compared to vitamin C (Wang et al., 2007). Many flavonoids identified in sand rice seeds and bran also exhibit antioxidant activity, which can protect DNA from 2,2′‐azobis(2‐amidinopropane) dihydrochloride‐induced damage and inhibit apoptosis in mammalian cells by upregulating Nrf2 and increasing Bcl‐2 expression (Xu et al., 2018).

Phenolic compounds have high structural stability, and the hydroxyl group on the benzene ring can very easily lose hydrogen ions; hence, phenolic compounds can show antioxidant activity as an electron donor (Chen et al., 2022). Quinoa grain contains numerous phenolic compounds, with ferulic acid and gallic acid being the dominant‐free phenols among seven quinoa varieties. Additionally, various phenolic acids and their derivatives in quinoa seeds contribute to their antioxidant activities (Hussain et al., 2021). Furthermore, quinoa exhibits antioxidant activity through its polysaccharides and peptides. Quinoa polysaccharide components demonstrate ABTS and DPPH free radical‐scavenging abilities, with a stronger affinity for ABTS free radicals (Tan et al., 2020; Teng et al., 2021). Ren et al. (2017) detected the antioxidant effects of the amino acid‐active peptide lunasin in quinoa and revealed that the ABTS^+^ free radical‐scavenging activity of lunasin was higher than its DPPH free radical‐scavenging activity.

Rutin, quercetin, and kaempferol‐3‐O‐rutinoside are the most abundant phenolic compounds in buckwheat and are present in free and bound forms. Furthermore, the antioxidant activity of free phenols is higher than that of the bound phenols in buckwheat (Zhu et al., 2019). Among the components contributing to the total antioxidant activity of buckwheat seeds, rutin plays the most prominent role (Lee et al., 2016). In vitro antioxidant tests have demonstrated strong antioxidant activity of rutin in various antioxidant systems, with its antioxidant capacity increasing with its concentration (Yang et al., 2008). The antioxidant activity of buckwheat extracts is associated with the extraction solvent and analysis method used. The methanol extract of buckwheat exhibits the highest antioxidant activity, whereas the acetone extract demonstrates the highest antioxidant activity when measured using the DPPH free radical removal method (Sun & Ho, 2005). Additionally, some common buckwheat proteins contain antioxidant peptides. Proteins isolated from buckwheat and hydrolyzed using alkaline proteases exhibit strong antioxidant abilities in different in vitro oxidation models (Jin et al., 2022). Among the reported buckwheat protein peptides with antioxidant activity, the tetrapeptide VFPW is the most effective free radical scavenger. In addition to the free radicals of the Phe (F) residues exerting antioxidant effects by trapping aromatic rings, the hydrophobic amino acid residues (V, L) in the proteolysate sequence also contribute to the antioxidant effect of the peptide through some unknown mechanism (Jin et al., 2022).

Sand rice seeds exhibit a total phenolic content of 163.01 mg gallic acid equivalents per 100 g of dry weight (mg GAE/100 g), which is lower than that of quinoa (167.2–308 mg GAE/100 g) and buckwheat seeds (518–657 mg GAE/100 g) but significantly higher than that of daily rice and millet. Sand rice also demonstrates a correspondingly elevated ability to scavenge ABTS^+^ and DPPH free radicals and reduce trivalent iron compared to rice and millet (Hussain et al., 2021; Wang et al., 2019; Zhu et al., 2019). Moreover, the total flavonoid content of sand rice coat (335.7 μg RE/g) is similar to that of soybeans, whereas the free flavonoid content of sand rice seeds (2.82 mg RE/g) surpasses that of colored quinoa grown in the Peruvian altiplano and rice, millet, wheat, and corn (Abderrahim et al., 2015; Wang et al., 2019; Xu et al., 2018). Therefore, the abundance of antioxidant components in sand rice holds significant benefits for individuals with oxidative stress‐related diseases.

#### 1.3.2 Anticancer activity

Phenolic compounds are secondary metabolites found in plants. Various phenolic compounds extracted from sand rice seeds, including protocatechuic and ferulic acids, play an important role in preventing cancer (Wang, Gong, et al., 2020; Wang, Wang, et al., 2020). Protocatechuic acid is cytotoxic to various cancer cells, including MCF‐7 cells (human breast cancer), A549 cells (lung cancer), and HeLa cells (cervical cancer) (Quinn et al., 2017). Ferulic acid has high free radical‐scavenging and antioxidant activities and prevents cancer through its anti‐mutation effect (Boz, 2015). The anticancer activity of phenolic acids has been reported to be related to the structure and molecular targets of the compounds, in which the number and location of phenolic hydroxyl groups play a key role (Saleem et al., 2022). Other flavonoids present in sand rice, specifically quercetin, exhibit anticancer activity. Quercetin induces cancer cell apoptosis, causes cell cycle stagnation, and can also prevent the migration of growth factor‐induced hepatocellular carcinoma cells by inhibiting the AKT signaling pathway (Yamada et al., 2020).

The polysaccharide component of quinoa has been demonstrated to have an inhibitory effect on MCF‐7 breast and SMMC7221 human liver cancer cells but not on normal cells (Hu et al., 2017). Shen et al. (2022) analyzed the physical, chemical, antioxidant, and anticancer properties of seed oil from three genotypes of quinoa (red, white, and black quinoa) and found that the darker the seed color, the more significant the growth inhibition effect of quinoa on HCT116 colon cancer cells. Amaranthin and apigenin II, present in the hypocotyl of quinoa, also inhibit cancer cell activity, though slightly (Ren et al., 2022). Many phenols and flavonoids extracted from quinoa leaves and seeds exhibit anticancer activity (Villacrés et al., 2022). For example, the main free phenols, ferulic and sorbic acids, in the seven quinoa varieties evaluated by Hussain et al. (2021) exert anticancer activity.

Buckwheat bran is rich in bioactive compounds. Different buckwheat shell extracts and in vitro experiments have revealed that buckwheat shell extracts can suppress various cancer cells, including human A549 cells, AGS cells (gastric adenocarcinoma), Hep‐3B cells (hepatocellular carcinoma), MCF‐7 cells, and HeLa cells; however, its anticancer effect gradually decreases with an increase in the concentration of the extract (Kim et al., 2007). Moreover, the polysaccharide components of buckwheat are cytotoxic to human liver cancer, breast cancer, prostate cancer, and other cancer cells, and they inhibit their growth (Zhu, 2020).

Similar to quinoa and buckwheat, the anticancer activity of sand rice is mainly achieved through its abundant bioactive substances (Zhao et al., 2021). However, the role of these bioactive substances in the anticancer effect of sand rice has not been studied extensively. Therefore, further research is required to promote the development and utilization of sand rice.

#### 1.3.3 Anti‐diabetic activity

Sand rice is ideal for improving blood sugar levels in individuals with diabetes. Compared to quinoa and buckwheat, sand rice contains a relatively elevated content of high‐resistance and slow digestible starches (Liu et al., 2015; Ren et al., 2020). The slow hydrolysis rate of sand rice starch lowers its glycemic index (Wang, Gong, et al., 2020; Wang, Wang, et al., 2020). In hyperglycemic rats fed on quinoa seeds for 2 weeks, blood glucose levels consistently decreased indicating that quinoa seeds contained substances with hypoglycemic effects (Alamri et al., 2023).

Takahama and Hirota (2010) have reported that rutin present in buckwheat binds with starch molecules to inhibit the catalytic rate of enzymes, thereby limiting starch digestion and reducing blood sugar levels. Lee et al. (2021) found that adding 10% Tartary buckwheat to the diet of mice with type 2 diabetes mellitus (T2DM) reduced their blood sugar and glycated hemoglobin levels. Thus, high rutin content of sand rice supports its potential as an anti‐diabetic agent.

Consistently, sand rice has shown potential anti‐glycemic activity in animal models and clinical trials (Bao et al., 2019; Bao & Han, 2016; Huang et al., 2015; Kang et al., 2020). Goto‐Kakizaki (GK) rats and KKAy mice are excellent models of spontaneous T2DM, with very similar etiology and pathogenesis as human T2DM (Huang et al., 2015; Kang et al., 2020). In GK rats with spontaneous T2DM that were administered agiophyllum oligosaccharides (AOS; 0.96, 0.48, and 0.24 g/kg) via gastric administration, insulin release was promoted, islet apoptosis was inhibited, islet function was improved, and random blood glucose levels were significantly reduced after 8 weeks of AOS administration (Bao & Han, 2016). Similarly, in KKAy mice administered sand rice extract (1.2, 0.6, and 0.3 g/kg) for 8 weeks continuously through the gastric route (Bao et al., 2019), the random blood glucose level of each mouse group decreased to varying degrees, glucose tolerance increased, and glycated serum protein levels and advanced glycosylation end products in serum decreased; furthermore, high‐dose drug treatment significantly increased the amount of liver glycogen synthesis. Sand rice extract was speculated to reduce blood sugar levels and insulin sensitivity by regulating the expression of islet signaling factors, including IRS2, PI3K, PAKT, GSK3β, and GLUT4.

Sand rice extract has shown anti‐glycemic effect in clinical settings (Zhao et al., 2021). Among 12 patients with hyperglycemia and five patients with diabetes who were administered sand rice ethanol extract daily for 3 weeks, only eight patients remained hyperglycemic, while the glycemic index of four out of the five patients with diabetes decreased significantly (Zhao et al., 2021). A strong two‐way correlation has been reported between T2DM and nonalcoholic fatty liver disease (NAFLD) (Li, Lietz, & Seal, 2021; Li, Ye, et al., 2021). The sensitivity of patients with type 2 diabetes to insulin is reduced, leading to insulin resistance, releasing free fatty acids (FFA), and increasing the possibility of FFA entering the liver. Sand rice extract lowers blood sugar levels, improves insulin resistance, and inhibits diabetes‐induced liver damage (Bao et al., 2022). Thus, sand rice extracts may be developed for treating T2DM‐NAFLD considering the connection between T2DM and NAFLD.

#### 1.3.4 Anti‐inflammatory activity

Sand rice extract inhibits the expression of inflammation‐related genes (Jiao et al., 2022). Lamb fat cells cultured in the presence of sand rice water extract showed decreased expression of inflammation‐related mediators, such as IL‐10, indicating the anti‐inflammatory ability of sand rice extract. In a study on hypoglycemia and diabetes‐induced liver and kidney damage in GK rats and db/db mice (an ideal animal model for studying T2DM), Bao and Ao (2021) found that AOS significantly reduced NF‐κB protein expression in the liver and kidney tissue of GK rats, reduced inflammatory cytokines, and played an anti‐inflammatory role. In the db/db mouse model, AOS reduced the infiltration of inflammatory cells in the liver of mice, alleviating liver damage (Bao & Ao, 2021).

Lipopolysaccharide (LPS)‐induced inflammation stimulates macrophages to produce inflammatory compounds. Ren et al. (2017) found that lunasin present in quinoa could dose‐dependently inhibit the LPS‐induced increase in NO, TNF‐α, and IL‐6, indicating its anti‐inflammatory effects. The isolated protein and saponin components of quinoa also exhibit anti‐inflammatory activities. Moreover, quinoa protein as well as glycosylated quinoa protein significantly inhibits the LPS‐induced release of NO (Teng et al., 2021). Chenopodin, an 11S globulin isolated from quinoa seeds, reduces carrageenan‐induced foot swelling and neutrophil aggregation in mice, showing anti‐inflammatory activity (Pompeu et al., 2022). Notably, the four saponin components, Q30, Q50, Q70, and Q90, isolated from quinoa dose‐dependently reduce NO production, inhibit inflammatory mediators such as TNF‐α and IL‐6, and exhibit anti‐inflammatory activity in RAW264.7 macrophages (Yao et al., 2014).

Long‐term inflammation can harm various organs in the human body, including the liver, brain, kidneys, and heart (Ge et al., 2022). Sand rice, quinoa, and buckwheat effectively regulate inflammatory responses. Sand rice has been used in Mongolian medicine; the Mongolian medicine preparation “Shahuang Capsule” can alleviate diabetes symptoms in mice, inhibit inflammation, and protect the liver and kidneys (Bao & Ao, 2021). Therefore, innovative formulations of sand rice can promote its wider use in clinical settings.

### 1.4 Food processing methods and their impact on grain nutrients

Pseudocereals are usually processed before consumption to obtain a better sensory quality. However, these processing methods can affect the contents of nutrients or bioactive mixtures in the grains. Therefore, during food development research on grains, it is important to consider the impact of different processing methods on food quality. The processing methods used for sand rice, quinoa, and buckwheat are described below.

**FIGURE 3:**
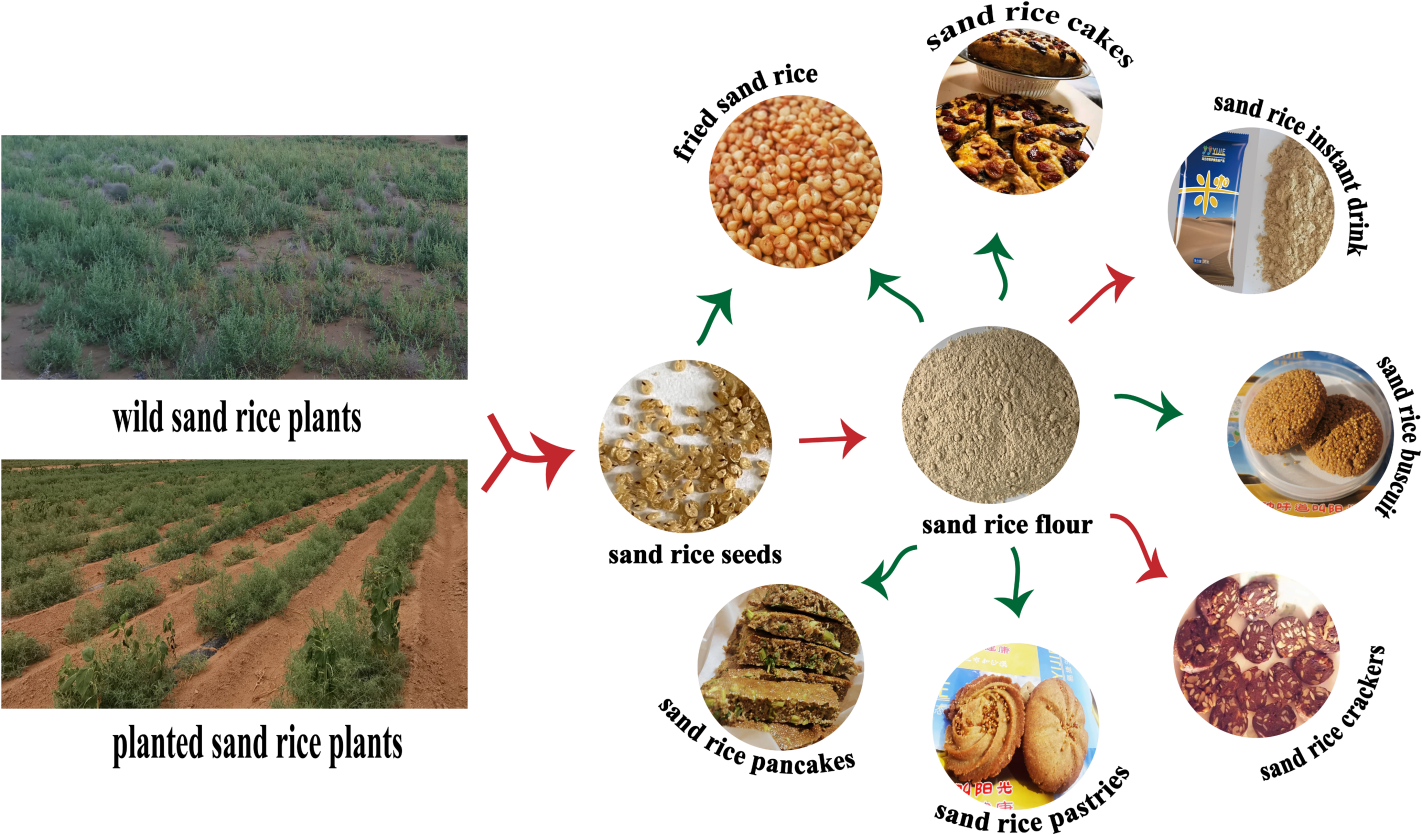
Possible novel sand rice‐based food products that can be included in human diet.

In China, the early Hexi people harvested sand rice, milled the sand rice seeds, and ate them like millets or used them as sand rice flour. Currently, sand rice is used in fried noodles, bean jelly, knife noodles, mutton noodles, and other snacks (Xu et al., 2020). After sand rice seeds are milled into a powder, they can be processed into new food products (Figure 3), such as ready‐to‐use drinks, cakes, biscuits, pastries, and sunflower pastries. Sand rice seeds can also be consumed directly as snacks after frying. A compound meal replacement powder with certain functional characteristics can be prepared by combining sand rice and stir‐fried white kidney bean powder. Furthermore, sand rice seeds can be fermented into yogurt, wine, and other products (Tian et al., 2022).

Quinoa flour is commonly used in food processing. Quinoa seeds are milled into meals and mixed with other grains or pseudocereal flours, such as buckwheat and wheat flours, to prepare bread, noodles, pancakes, biscuits, and other foods through traditional baking methods (Tian et al., 2022). In some regions, quinoa seeds are stewed and used for making salads, soups, and other foods, and they can also be consumed as vegetables (Kuktaite et al., 2022; Sezgin & Sanlier, 2019).

Milling is a traditional processing method used for buckwheat or Tartary buckwheat. It is used widely to produce porridge, pancakes, bread, noodles, and other foods using buckwheat dough. Furthermore, fermented buckwheat is used to produce vinegar, yogurt, black soy sauce, and alcohol (Bender & Schönlechner, 2021). Tartary buckwheat seeds can be used for developing various beverages. The stems of golden buckwheat are also used to develop commercial drugs owing to their medicinal properties (Sinkovič et al., 2022).

Adding sand rice flour during rice noodle preparation can significantly improve the protein, fat, and cellulose content of the mixed powder and reduce carbohydrate content (Wei et al., 2022). In addition to the beneficial substances, sand rice, and quinoa also contain anti‐nutrient saponins. The content of saponins in sand rice seeds is low at 5.81 g/100 g but higher than that in quinoa seeds (0.1%–5.0%) (Chaudhary et al., 2023; Zhao et al., 2021). Saponins have anti‐inflammatory, antioxidant, analgesic, antibacterial, antiviral, and other positive effects, but they can also affect the absorption of vitamins and some minerals. Therefore, special treatment is required before consuming seeds to remove saponins (Chaudhary et al., 2023). Milling or other post‐harvesting processes can effectively remove saponins from the seed skin of quinoa. Wang, Gong, et al. (2020); Wang, Wang, et al. (2020) studied the effect of peeling on the physical and chemical properties of quinoa and compared them with those of full and traditional flour. Peeling quinoa before milling reduced saponin, phytic acid, ash, and insoluble dietary fiber contents in quinoa powder. Moreover, the total phenol and mineral contents were higher than those in traditional flour, and protein, starch, and soluble dietary fiber contents were higher than those in the powder prepared from unpeeled quinoa. Thus, peeling is an effective processing method for improving the nutrient content of quinoa flour.

Buckwheat is generally used to develop gluten‐free food after the peeling step (Martínez‐Villaluenga et al., 2020). Processing methods such as milling, washing, and heat treatment are usually employed in producing quinoa‐ and buckwheat‐based foods. Tumpaung et al. (2021) compared the effects of milling and washing on the nutritional composition of quinoa seeds. Processing affects the content of some minerals and significantly reduces the ash content of quinoa grains. Additionally, the number of grinding steps and different washing solutions may decrease the lipid content of quinoa. Different grinding methods have different effects on buckwheat grains. Yu et al. (2018) investigated the effects of high‐speed universal grinding, wet milling, and stone milling on the physical and chemical properties of buckwheat. Among these, wet milling relatively preserved the buckwheat particle structure while maintaining the starch structure and content; however, it significantly reduced the total flavonoid content and water adhesion property.

During sand rice food processing (Wang et al., 2019), boiling treatment increases the total phenol content of the water phase‐ and solid‐phase residues, whereas the flavonoid content is slightly reduced in the water phase but increases in the solid residues. Thus, cooking promotes the release of phytochemicals from sand rice seeds. The production of sand rice composite powder by applying twin‐screw extrusion technology has been investigated (Li et al., 2019). Squeezing leads to a darkening of the color of the sand rice composite powder, significantly reducing its peak, valley, and final viscosities as well as the breakdown, setback, and attenuation values. However, enzyme pretreatment significantly improves the physical and chemical properties of sand rice composite powder (Li et al., 2019). By reducing the retrogradation value, the shelf life of sand rice can be prolonged, which is beneficial for storage purposes.

Among different heat treatment methods, boiling significantly reduces certain water‐soluble minerals and polyphenols in quinoa, whereas baking treatment considerably reduces the total phenol content and antioxidant properties (Mhada et al., 2020; Nickel et al., 2016). However, cooking methods can help preserve these substances. Pressurized cooking has shown promising results in significantly improving the total phenol content and antioxidant ability. After pressurized heating, the content of plant active substances and saponins in quinoa remains similar to that in raw quinoa while maintaining the complete form of the quinoa grains (Mhada et al., 2020; Nickel et al., 2016). Baking significantly increases the total phenol content of ordinary buckwheat but reduces the total flavonoid content (Ma et al., 2020). Baking also improves the protein and fat contents of buckwheat. Improved nutritional profile and antioxidation ability of buckwheat make it more suitable for developing baked goods. Heating and pressure treatment can preserve the lipid content in buckwheat while inhibiting lipase activity, thus extending the storage duration of buckwheat products (Wang, Gong, et al., 2020; Wang, Wang, et al., 2020). Moreover, the adoption of improved extrusion and cooking technologies to process buckwheat‐based food alters the particle shape of buckwheat starch, changes its structure, and improves the degree of gelatinization, allowing its utilization as thickeners in various applications (Cheng et al., 2020).

Processing methods effectively improve the nutritional composition of quinoa and buckwheat. Compared to quinoa and buckwheat, the processing of sand rice mostly enhances its bioactive substances and changes its physicochemical properties. However, the effects of sand rice food processing on its nutritional value have not been studied extensively compared to effects on quinoa and buckwheat. Nonetheless, employing various methods to improve the sensory quality of sand rice‐based foods has proven effective. Further research on quinoa and buckwheat can facilitate sand rice processing, develop sand rice‐based foods, and expand their consumption in the future.

## 2 THE POTENTIAL FOR THE PRODUCTION AND DEVELOPMENT OF SAND RICE

Functional food development is a popular trend in the food industry. With people's in‐depth understanding of the nutritional and medicinal value of sand rice, the development of functional foods may become an important direction for sand rice‐based food in the future. The protein content characteristics of sand rice provide a theoretical basis for the development of gluten‐free food. The crude fiber content in sand rice also makes it a functional food and meal substitute for a balanced diet and weight control (Zhao et al., 2021). Moreover, as mentioned above, the wide pharmacological activities of bioactive substances in sand rice also support the development of sand rice extract as a drug for treating diabetes, hyperlipidemia, and other diseases. In addition to basic food processing, sand rice flour is added to food recipes to improve the flavor and texture of food (Wei et al., 2022). However, wild sand rice has low yield and some unfavorable agronomic traits; hence, domestication of sand rice is necessary. In recent years, the feasibility of sand rice domestication has been analyzed. The breeding process of sand rice can be accelerated by screening for excellent traits for introduction and cultivation, and by using physicochemical mutagenesis to improve the yield and achieve the goal of sand rice domestication (Ma et al., 2021; Zhao et al., 2016). According to an artificial cultivation test, it is estimated that the yield of artificial cultivation of sand rice can reach 950 kg/hm^2^ (Zhang et al., 2019). Thus, sand rice has great production potential to be incorporated into bread, soup, and other sand rice‐based foods to supplement human daily nutrition. Additionally, simple food processing methods may be applied to retain or improve the nutritional value or physiological activity in sand rice.

### 2.1 Conclusion and future prospects

This review comprehensively evaluates the nutritional composition, health benefits, and food processing methods of sand rice compared to those of quinoa and buckwheat. Sand rice is characterized by high‐protein, high‐quality fatty acids, and highly resistant starch contents, which makes it an important and novel source of functional food that also meets the sensory and nutritional needs of modern consumers. Phenols, including protocatechuic, ferulic, and chlorogenic acids, and flavonoids, including quercetin and rutin, are the main bioactive compounds in sand rice. These bioactive compounds can improve blood sugar levels and effectively prevent chronic diseases caused by oxidative stress. Furthermore, sand rice extracts and some active compounds exhibit therapeutic potential for treating cancer, inflammation, and diabetes. However, only some scholars at home and abroad have studied the chemical composition of sand rice, and the relationship between the specific chemical composition and biological activity is not yet clear. There is relatively little research on the efficacy and mechanism of its bioactive substances. Therefore, to further understand the quality of sand rice and develop and utilize it more comprehensively and effectively, it is necessary to conduct in‐depth research.

In addition, significant progress has been made in developing sand rice for producing functional foods and in utilizing sand rice as an auxiliary material in composite food products to enhance sensory quality, nutritional profile, and functionality. The production of sand rice drinks through fermentation technology has also garnered significant attention. The gluten‐free nature of sand rice makes it suitable for developing gluten‐free products and a food source for patients with celiac disease. However, the processing technology requires further development and improvement. Thus, promoting the maximum utilization of sand rice grains in food industry and their consumption to improve human health is an important area of sand rice research in the future.

## AUTHOR CONTRIBUTIONS

**Xiaofan Yang:** Data curation (lead); formal analysis (lead); investigation (lead); writing – original draft (lead). **Liuyang Xiao:** Data curation (supporting); investigation (supporting). **Wenting Fu:** Data curation (supporting); investigation (supporting). **Zhaojun Wei:** Resources (supporting); writing – review and editing (supporting). **Lihong Han:** Funding acquisition (supporting); project administration (supporting); supervision (lead); writing – review and editing (supporting).

## CONFLICT OF INTEREST STATEMENT

All authors have declared no potential conflicts of interest.

## Data Availability

Data are openly available in a public repository.
